# Echocardiographic Findings in Small‐Breed Dogs With Myxomatous Mitral Valve Disease and the Severity of Mitral Regurgitation, Heart Size and Clinical Signs Myxomatous Mitral Valve Disease in Dogs

**DOI:** 10.1002/vms3.70922

**Published:** 2026-06-15

**Authors:** Boshra Elyasi

**Affiliations:** ^1^ Department of Clinical Science Faculty of Specialized Veterinary Sciences Science and Research Branch Islamic Azad University Tehran Iran

**Keywords:** Dog, Doppler echocardiography, mitral disease, valvular

## Abstract

**Background:**

The main goal of this study was to identify various brightness, motion and Doppler echocardiographic variables in small‐breed dogs with myxomatous mitral valve disease.

**Animals:**

Sixty client‐owned small‐breed dogs with mitral regurgitation murmurs.

**Methods:**

Echocardiography was performed in brightness, motion and Doppler modality from the right parasternal view. On the basis of thoracic radiographs, the dogs were categorized into two groups: those with cardiomegaly and those without cardiomegaly. Additionally, on the basis of the jet area signal to left atrium ratio in colour Doppler echocardiography, the canines were divided into three groups: mild, moderate and severe mitral regurgitation. Furthermore, the dogs were classified into preclinical and clinical groups on the basis of the presence or absence of clinical signs, and various variables were compared across these groups.

**Results:**

This difference was statistically significant and more prevalent in intact dogs than in spayed/neutered dogs. Significant differences were observed in variables including LAmax, LA:Ao, LVIDd, LVIDs, VMA, VMG, VMV, GMV, VMA‐GMVT, LVOT and GLVOT in dogs with myxomatous mitral valve disease of varying mitral regurgitation severity and between normal and enlarged hearts. Significant differences were also found in the FS, GMV, VLVOT and GLVOT variables between the clinical and preclinical groups. A moderate and statistically significant correlation was observed between LA:Ao and VMA, VMV and LVOT. Weak and mostly insignificant correlations were found between the Doppler and motion‐mode variables.

**Conclusions:**

Varying mitral regurgitation severity, heart size and the presence of clinical signs can significantly affect certain brightness, motion and Doppler echocardiographic variables in dogs with myxomatous mitral valve disease (valvular heart disease).

## Graphical abstract

Myxomatous mitral valve disease (MMVD) is the most common acquired cardiac disorder in small‐breed dogs and is characterized by progressive mitral regurgitation that can lead to cardiac enlargement and clinical signs of heart failure. Accurate assessment of structural and hemodynamic changes using two‐dimensional, M‐mode and Doppler echocardiography is essential for early detection, staging and monitoring of disease progression in affected dogs.

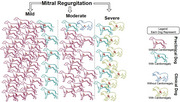

**Abbreviations**
Ao: aorta dimension
ARJ:LLA: jet area signal to left atrium ratio
EF: ejection fraction
FS: fractional shortening
GA: pressure gradient of aorta
GLVOT: pressure gradient of left ventricle outflow tract
GMA: pressure gradient of mitral flow wave at atrium side
GMV: pressure gradient of mitral flow wave at ventricle side
IVSd: interventricular septum thickness during the diastole
IVSs: interventricular septum thickness during the systole
LAmax: left atrial dimension
LVIDs: left ventricular internal dimension at end‐diastole
LVIDs: left ventricular internal dimension at end‐systole
LVPWd: left ventricular posterior wall thickness at end‐diastole
LVPWs: left ventricular posterior wall thickness at end‐systole
MMVD: myxomatous mitral valve disease
MR: mitral regurgitation
VA: peak velocity of aorta
VLVOT: peak velocity of left ventricle outflow tract
VMA: peak velocity of mitral flow wave at atrium side
VMV: peak velocity of mitral flow wave at ventricle side

## 1 Introduction

Cardiovascular diseases are among the leading causes of mortality and disability worldwide. Myxomatous mitral valve disease (MMVD) is the most common acquired heart disease in dogs (Keene et al. 2019; Mattin et al. 2015; Svensson et al. 2024), particularly in small‐breed dogs (Burchell and Schoeman 2014; Chetboul and Tissier 2012). Initially, this disease progresses silently without clinical signs, but over time, it leads to changes in heart size and, in severe cases, may result in death (Borgarelli and Buchanan 2012; O'Brien et al. 2021). For this reason, MMVD is considered the leading cause of heart disease‐related mortality in dogs (Häggström et al. 2009) and one of the primary causes of heart failure and death in humans (Levine et al. 2015).

This disease is characterized by MR, which results in left atrial and ventricular enlargement (Ljungvall 2024) in advanced stages, volume overload, left atrial and left ventricular remodelling, chordal rupture and even congestive heart failure (Bagardi et al. 2022).

Various tools are used to diagnose heart diseases, with echocardiography being one of the most effective methods (Chetboul and Tissier 2012; Klein et al. 2022). This technique allows for the evaluation of cardiac structures via different modalities, including brightness, colour and Doppler imaging (Serres et al. 2008). Colour Doppler imaging can detect even mild MR (Ljungvall 2024). Additionally, Doppler modalities provide objective haemodynamic data, such as velocity and pressure, which are essential for quantifying ventricular function and assessing the severity of valve lesions (Adham Esfahani et al. 2019; Harris and Kuppurao 2016).

However, despite numerous studies, an accurate understanding of the hemodynamic changes in the hearts of dogs with MMVD remains incomplete. This study was therefore conducted to identify various echocardiographic variables in these patients and to determine the severity of these changes across different clinical, radiographic and MR severity classifications in small‐breed dogs. Additionally, this study included an epidemiological evaluation of the age, sex, weight and breed of the dogs.

## 2 Materials and Methods

### 2.1 Animals

This study was conducted on small‐breed dogs with owners referred to XXXXXX veterinary hospital in XXX, XXX, between 2022 and 2024. The dogs were examined by a small animal internal medicine specialist via a Littmann stethoscope, and those with mitral regurgitation murmurs were selected for the study. Dogs that were receiving cardiac treatment or who were diagnosed with conditions such as neoplasia, anaemia, kidney failure, respiratory disorders or endocrine diseases were excluded.

**FIGURE 1:**
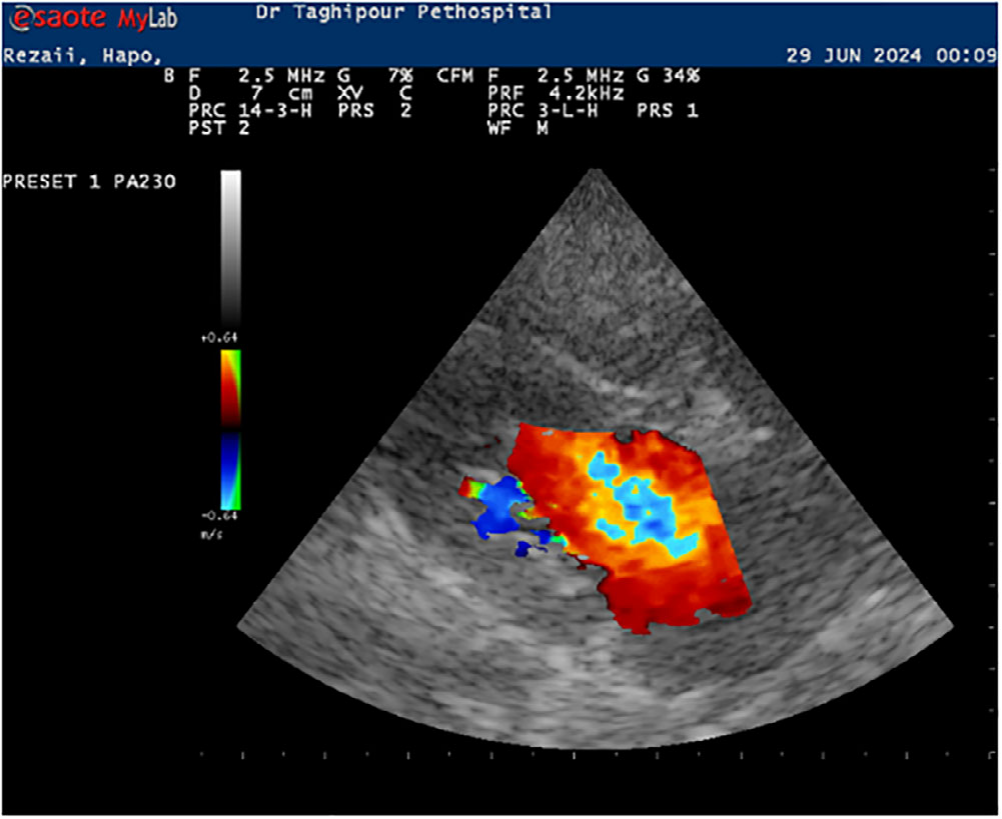
Echocardiographic image of a small‐breed dog with myxomatous mitral valve disease (MMVD). Right parasternal long‐axis four‐chamber view using Doppler modality, demonstrating mitral regurgitation (MR).

**FIGURE 2:**
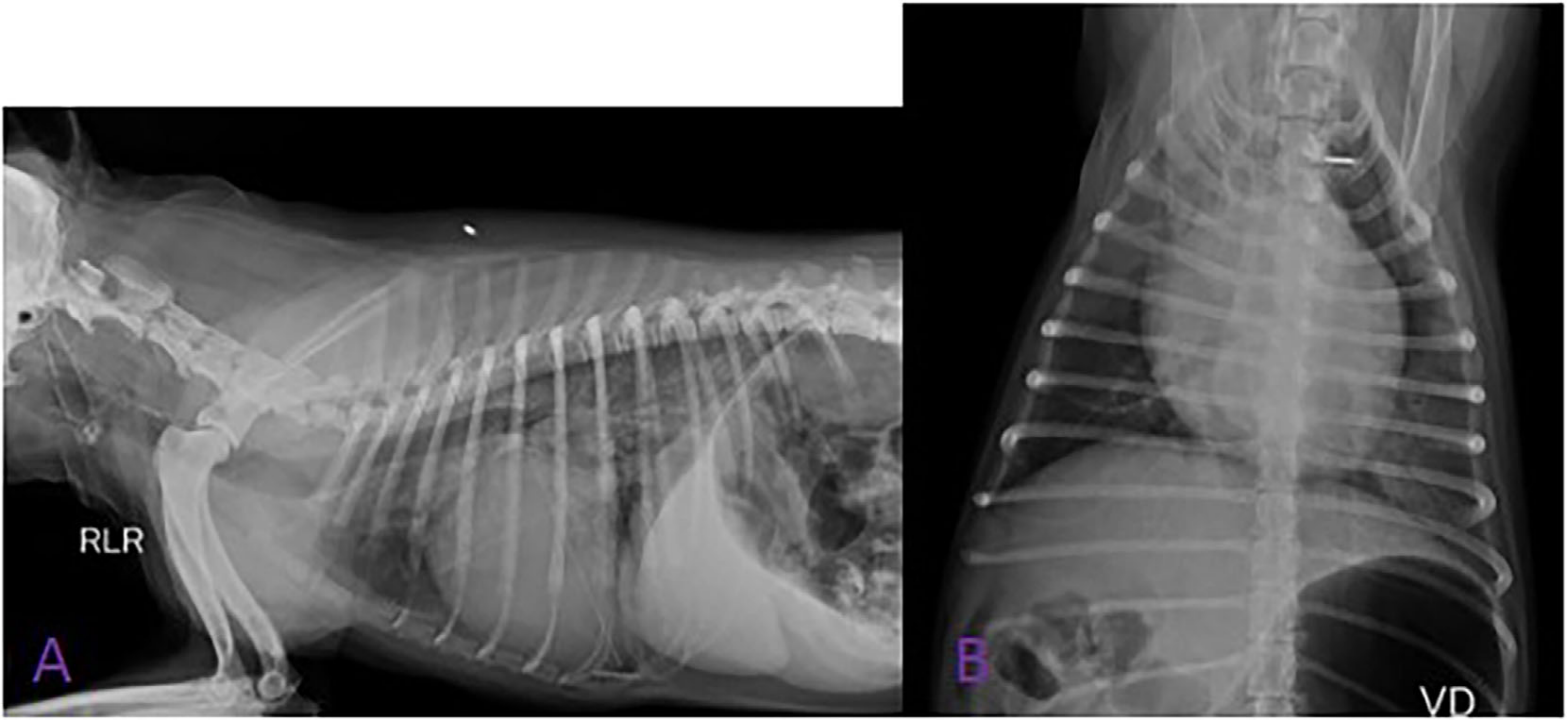
Radiographic images of a small‐breed dog with myxomatous mitral valve disease (MMVD) showing cardiomegaly. (A) Right lateral recumbency. (B) Left lateral recumbency.

After animal selection, echocardiography was performed by a board‐certified radiologist to confirm MMVD. Disease was confirmed if a mosaic mitral valve pattern was observed via colour Doppler imaging (Figure 1). Dogs diagnosed with other cardiac diseases were excluded. Thoracic radiographs were then obtained via a digital x‐ray machine (Konica, Japan) in right lateral recumbency and ventrodorsal views. On the basis of the criteria outlined by Bahr (2018), the hearts of the dogs were classified as either having cardiomegaly or being normal (Figure 2).

### 2.2 Echocardiographic Measurements

Echocardiography was performed via a MyLab 40 device (Esaote, Italy) with cardiac phased‐array transducers (frequency 3.5 MHz) by a board‐certified radiologist, following the standard protocol described by Bonagura and Schober (2009).

**FIGURE 3:**
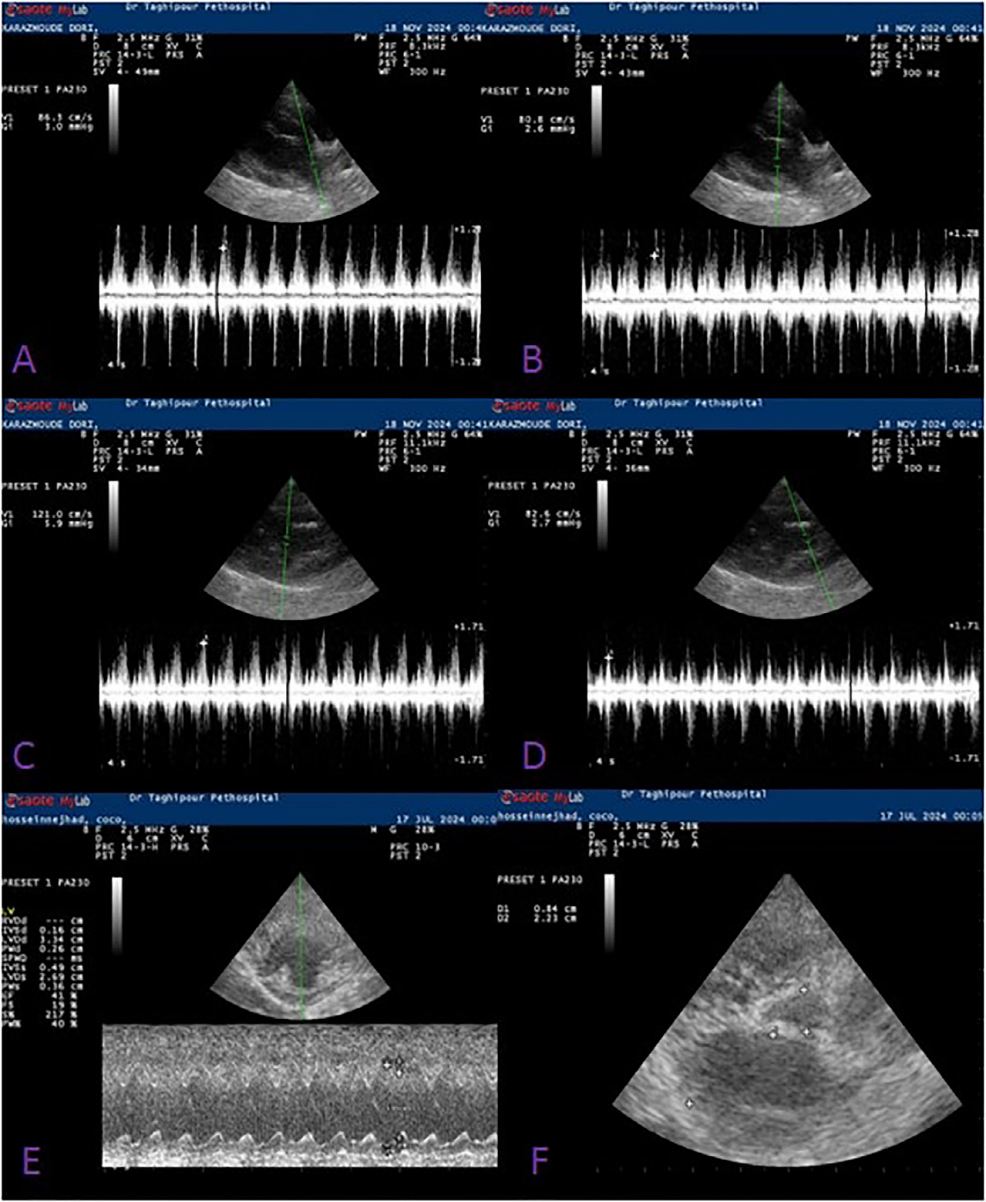
Echocardiographic images of a small‐breed dog with myxomatous mitral valve disease (MMVD). (A) Right parasternal long‐axis four‐chamber view, Doppler modality, showing peak velocity of mitral inflow at the atrial side. (B) Right parasternal long‐axis four‐chamber view, Doppler modality, showing peak velocity of mitral inflow at the ventricular side. (C) Right parasternal long‐axis five‐chamber view, Doppler modality, showing peak velocity of left ventricular outflow tract (LVOT). (D) Right parasternal long‐axis five‐chamber view, Doppler modality, showing peak velocity of the aorta. (E) Right parasternal short‐axis mushroom‐level view, motion (M‐mode) modality. (F) Right parasternal short‐axis aortic‐root view, brightness (B‐mode) modality, showing left atrium (LA) and aorta (Ao) diameters.

In the right parasternal, long‐axis at the view four‐chamber level, with the Doppler sample volume positioned at the anterior aspect of the mitral valve leaflets, mitral inflow velocity (atrium side) (VMA) and the corresponding pressure gradient (GMA) were measured. Subsequently, the Doppler sample volume was repositioned to the posterior aspect of the mitral valve, and mitral inflow velocity (ventricle side) (VMV) and pressure gradient (GMV) were recorded at this location as well (Figure 3A,B) (Boon 2011).

In the right parasternal, long‐axis view at the five‐chamber level, the Doppler sample volume was positioned at the level of the left ventricular outflow tract (LVOT) and LVOT velocity (VLVOT), along with the corresponding pressure gradient (GLVOT) were measured. Subsequently, in the same view, the sample volume was repositioned at the proximal portion of the aorta, where aortic flow velocity (VA) and the associated pressure gradient (GA) were recorded (Figure 3C,D) (Boon 2011).

In the right parasternal, short‐axis view at the level of the papillary muscles (mushroom level) using M‐mode echocardiography, measurements were obtained according to standard guidelines. First, the interventricular septal thickness in diastole (IVSd) was measured. Subsequently, the left ventricular internal diameter in diastole (LVIDd) was recorded in the same alignment, followed by measurement of the left ventricular free wall thickness in diastole (LVPWd). The cursor was then evaluated during systole to measure the interventricular septal thickness in systole (IVSs), the left ventricular internal diameter in systole (LVIDs) and finally the left ventricular free wall thickness in systole (LVPWs). Fractional shortening (FS) and ejection fraction (EF) were subsequently calculated using the Teichholz method (Figure 3E) (Boon 2011).

Using the short‐axis aortic root view in B‐mode, LAmax, Ao and the LA:Ao ratio were measured. For this method, the aortic diameter was measured at the level of the commissure between the non‐coronary and right coronary cusps. Subsequently, the left atrial diameter was obtained by drawing a linear measurement from the junction of the non‐coronary cusp to the left coronary cusp of the aortic valve and recording the maximal left atrial dimension (Figure 3F) (Boon 2011).

### 2.3 Classification of the Enrolled Patients

#### 2.3.1 Mitral Regurgitation Severity: Using a Semiquantitative Assessment

Mitral regurgitation severity was assessed using a semiquantitative approach similar to that described by Muzzi et al. (2003). The area of the regurgitant jet (ARJ) was measured at the site of the mosaic colour flow pattern extending from the mitral valve into the left atrium during systole using colour Doppler imaging. The left atrial area (LLA) was also determined. All measurements were obtained from the right parasternal long‐axis view at the four‐chamber level. Subsequently, the ARJ/LLA ratio was calculated. Based on the ARJ/LLA ratio, dogs were classified as having mild (<–30%), moderate (–30% to 70%) or severe (> 70%) mitral regurgitation

#### 2.3.2 Heart Size

Based on the radiographic measurements, the dogs were divided into two groups: those with cardiomegaly and those without cardiomegaly.

#### 2.3.3 Clinical Signs

Dogs were grouped into preclinical and clinical categories on the basis of clinical signs, including difficulty breathing, exercise intolerance, weakness and coughing.

### 2.4 Statistical Analysis

Statistical analysis was performed via SPSS software (version 24). The Kolmogorov–Smirnov test was used to evaluate data normality. Chi‐square tests were applied for frequency comparisons, ANOVA with Tukey post hoc tests was used for comparing means across three groups, and independent *t*‐tests were applied for comparing means between two groups. Correlations between echocardiographic variables were analysed via scatter plots and Pearson's correlation coefficient. A significance level of *p* < 0.05 was considered statistically significant.

## 3 Results

### 3.1 Assessment of Data Distribution

The results of the Kolmogorov‒Smirnov test indicated that the data were normally distributed (*p* > 0.05). Therefore, parametric tests were used for subsequent analyses.

### 3.2 Animal Characteristics

The findings revealed that MMVD was significantly more prevalent in intact dogs than in spayed/neutered dogs (*p* = 0.02). The Terrier, Pomeranian, Shih Tzu and Terrier Mix breeds had the highest prevalence of MMVD (*p* = 0.00). Notably, these breeds are among the most popular in Iran and, therefore, represent the majority of referrals. No differences in MMVD incidence were observed based on age (*p* = 0.34), weight (*p* = 0.28) or sex (*p* = 0.76) (Table 1).

**TABLE 1:** Signalment characteristics of 60 small‐breed dogs diagnosed with myxomatous mitral valve disease (MMVD).

	Group	Frequency (percent)	*p* value
Age (years)	≤ 4	12 (20%)	0.34^a^
	> 4–≤ 8	21 (35%)	
	> 8–≤ 12	13 (21.7%)	
	> 12	14 (23.3%)	
Neuter status	Intact	21 (35%)	0.02^b^
	Neutered/spayed	39 (65%)	
Body weight (kg)	≤ 4	15 (25%)	0.28^a^
	> 4–≤ 8	25 (41.7%)	
	> 8–≤ 12	20 (33.3%)	
Gender	Female	29 (48.3%)	0.76^b^
	Male	31 (51.7%)	
Breed	Terrier	11 (18.3%)	0.00^a^
	Pomeranian	12 (20%)	
	Shih Tzu	12 (20%)	
	Chihuahua	3 (5%)	
	Terrier mix	11 (18.3%)	
	Spitz	3 (5%)	
	Pekingese	5 (8.3%)	
	Maltese	1 (1.7%)	
	Dachshund	1 (1.7%)	
	Poodle	1 (1.7%)	

*Note*: Data are presented as the number of dogs (percentage of total dogs in each group).

^a^ *p* value calculated using one‐way ANOVA for three‐group comparisons.

^b^ *p* value calculated using *t*‐test for two‐group comparisons.

### 3.3 Group Characteristics

**FIGURE 4:**
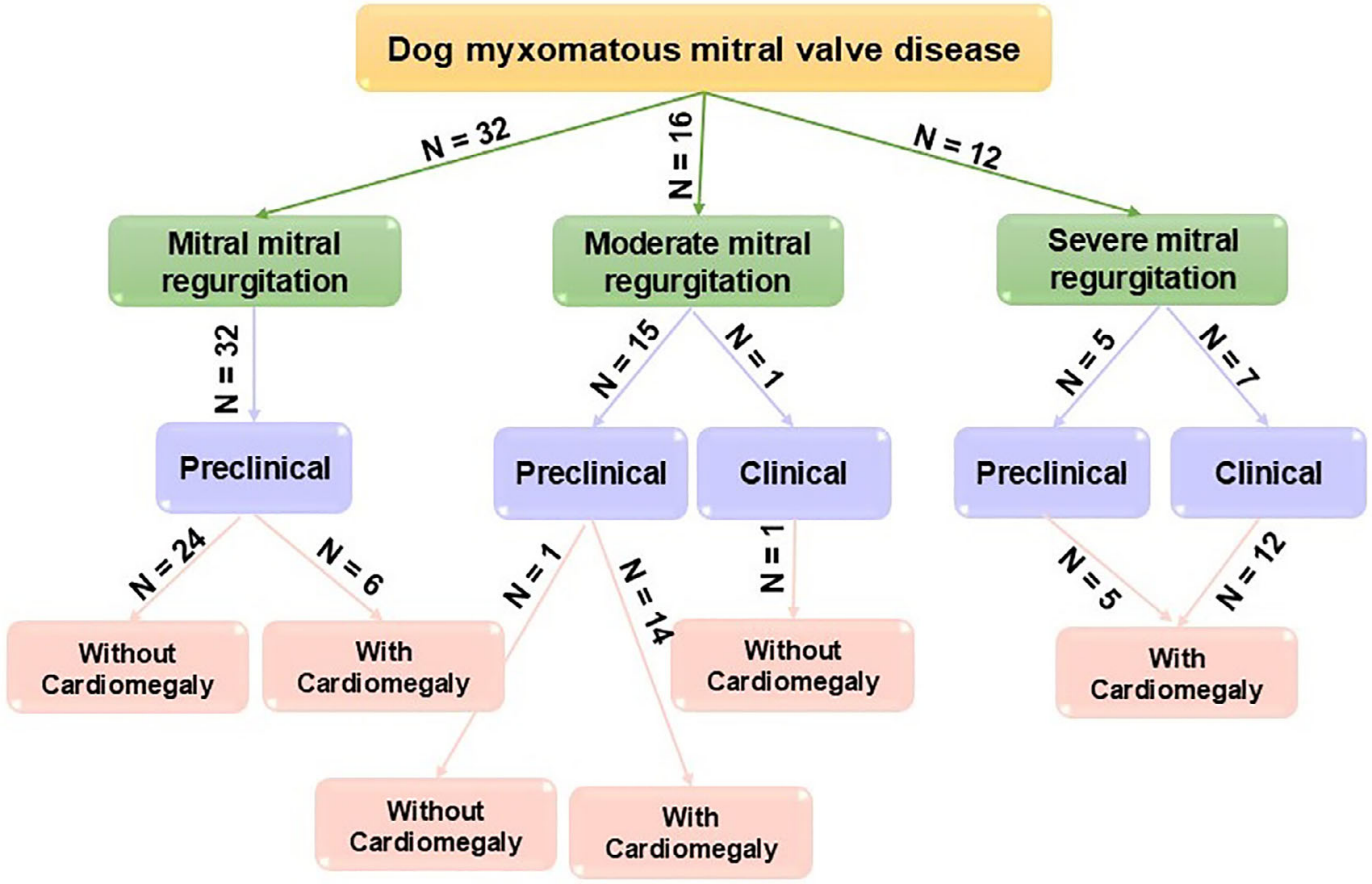
Integration of classifications in 60 small‐breed dogs with myxomatous mitral valve disease (MMVD) based on the severity of mitral regurgitation (MR), cardiac enlargement and the presence of clinical signs. N: number of cases.

As shown in Figure 4, in the mild MR group, the highest frequency was observed in dogs without cardiomegaly and in the preclinical group. In the moderate MR group, the highest frequency was observed in dogs with cardiomegaly, followed by those in the preclinical group. In the severe MR group, all dogs had cardiomegaly, with the highest frequency belonging to the clinical group. The highest frequency of clinical cases was observed in the severe MR group.

### 3.4 Echocardiographic Variables

As shown in Table 2, the mean values for LAmax, LVIDd, VMA‐VMV and VLVOT in the severe MR group were significantly greater than those in the mild and moderate groups. However, no differences were observed between the mild and moderate groups. The mean values for LA:Ao and VMA showed a decreasing trend from the severe to the moderate to the mild groups, with statistically significant differences. The mean values for LVIDs, GMA, VMV, GMV and GLVOT were significantly greater in the severe MR group than in the mild MR group, but no differences were observed between the other groups.

**TABLE 2:** Echocardiographic variables in 60 small‐breed dogs with myxomatous mitral valve disease (MMVD), grouped by mitral regurgitation severity and cardiac enlargement.

	Severity of mitral jet (*n* = 60)				Heart size (*n* = 60)		
Group variable	Mild (1) (*n* = 32)	Moderate (2) (*n* = 16)	Severe (3) (*n* = 12)	*p* value (ANOVA)^a^	Normal (*N* = 28)	Cardiomegaly (*N* = 32)	*p* value (*t*‐test)^b^
LAmax (cm)	1.45 (0.78–2.12)	1.71 (1.18–3.03)	2.10 (1.51–3.08)	1–2: 0.10 1–3: 0.00 2–3: 0.03	1.40 (0.78–2.05)	1.87 (1.12–3.08)	0.004
Ao (cm)	1.23 (0.66–1.54)	1.17 (0.91–1.51)	1.19 (0.84–1.44)	0.64	1.23 (0.66–1.52)	1.19 (0.71–1.54)	0.44
LA: Ao	1.17 (0.84–1.67)	1.47 (0.93–2.72)	1.77 (1.22–2.65)	1–2: 0.01 1–3: 0.00 2–3: 0.05	1.13 (0.84–1.43)	1.58 (1.02–2.72)	0.000
IVSd (cm)	0.40 (0.24–0.55)	0.37 (0.16–0.55)	0.34 (0.16–0.56)	0.17	0.39 (0.24–0.55)	0.36 (0.16–0.56)	0.20
LVIDd (cm)	2.49 (1.21–3.49)	2.74 (1.99–3.59)	3.24 (2.10–4.87)	1–2: 0.36 1–3: 0.001 2–3: 0.08	2.44 (1.55–3.49)	2.94 (1.21–4.87)	0.003
LVPWd (cm)	0.39 0.26–0.59)	0.37 (0.18–0.58)	0.38 (0.21–0.56)	0.66	0.40 (0.27–0.55)	0.37 (0.18–0.59)	0.33
IVSs (cm)	0.67 (0.34–0.93)	0.59 (0.39–0.86)	0.67 (0.41–0.89)	0.21	0.68 (0.47–0.93)	0.62 (0.34–0.900	0.12
LVIDs (cm)	1.64 (0.93–2.41)	1.90 (1.40–2.56)	2.15 (1.29–3.06)	1–2: 0.11 1–3: 0.002 2–3: 0.26	1.61 (1.06–2.41)	1.99 (0.93–3.06)	0.001
LVPWs (cm)	0.65 (0.41–0.86)	0.71 (0.47–2.69)	0.68 (0.36–0.89)	0.82	0.66 (0.41–0.86)	0.69 (0.36–2.69)	0.67
EF (%)	63.59 (49–77)	59.81 (43–73)	63.25 (41–80)	0.34	64.10 (49–77)	61.12 (41–80)	0.18
FS (%)	33.28 (23–43)	30.75 (20–40)	33.58 (19–47)	0.33	33.35 (24–43)	32.06 (19–47)	0.40
VMA (cm/s)	57.21 (38.70–86.30)	71.22 (43.10–111.50)	92.76 (64.30–128.30)	1–2: 0.007 1–3: 0.000 2–3: 0.001	55.30 (38.70–71.40)	79.22 (46.50–128.30)	0.000
GMA (mmHg)	1.45 (0.60–3)	2.47 (0.70–11.80)	3.52 (0.50–6.60)	1–2: 0.10 1–3: 0.001 2–3: 0.21	1.28 (0.60–2.10)	2.88 (0.50–11.80)	0.000
VMV (cm/s)	54.29 (39.80–80.80)	61.01 (31–81.10)	68.69 (35.40–121.40)	1–2: 0.24 1–3: 0.008 2–3: 0.30	53.64 (39.80–71.40)	63.62 (31–121.40)	0.007
VMA‐VMV (cm/s)	2.92 (−18.80 to 15.50)	10.20 (−23.20 to 36.50)	24.07 (−32.90 to 37.90)	1–2: 0.24 1–3: 0.00 2–3: 0.04	1.65 (−18.80 to 15.50)	15.60 (−32.90 to 37.90)	0.001
GMV (mmHg)	1.23 (0.60–2.60)	1.58 (0.40–3.00)	1.94 (0.50–3.80)	1–2: 0.19 1–3: 0.005 2–3: 0.31	1.20 (0.60–2.10)	1.70 (0.40–3.80)	0.005
VA (cm/s)	65.90 (41.30–97.40)	69.69 (52.20–99.60)	69.34 (45.40–104.00)	0.73	66.15 (41.30–97.40)	68.87 (44.30–104.00)	0.55
GA (mmHg)	1.91 (0.70–3.90)	1.98 (0.90–4.00)	1.99 (0.80–4.30)	0.96	1.91 (0.70–3.90)	1.98 (0.80–4.30)	0.79
VLVOT (cm/s)	59.78 (42.80–89.20)	61.86 (34.30–90.00)	72.56 (44.30–99.30)	1–2: 0.86 1–3: 0.01 2–3: 0.09	58.97 (42.80–82.00)	66.32 (34.30–99.30)	0.03
GLVOT (mmHg)	1.46 (0.70–3.20)	1.60 (0.70–3.20)	2.12 (0.80–3.90)	1–2: 0.81 1–3: 0.02 2–3: 0.13	1.41 (0.70–2.80)	1.82 (0.70–3.90)	0.02

*Note*: Values are presented as mean (minimum–maximum).

Abbreviations: Ao, aorta dimension; EF, ejection fraction; FS, fractional shortening; GA, pressure gradient of aorta; GLVOT, pressure gradient of left ventricle outflow tract; GMA, pressure gradient of mitral flow wave at atrium side; GMV, pressure gradient of mitral flow wave at ventricle side; IVSd, interventricular septum thickness during the diastole; IVSs, interventricular septum thickness during the systole; LAmax, left atrial dimension; LVIDs, left ventricular internal dimension at end‐diastole; LVIDs, left ventricular internal dimension at end‐systole; LVPWd, left ventricular posterior wall thickness at end‐diastole; LVPWs, left ventricular posterior wall thickness at end‐systole; *N*, number of cases; VA, peak velocity of aorta; VLVOT, peak velocity of left ventricle outflow tract; VMA, peak velocity of mitral flow wave at atrium side; VMV, peak velocity of mitral flow wave at ventricle side.

^a^ *p* values calculated using one‐way ANOVA for three‐group comparisons and independent *t*‐test. Post‐hoc pairwise comparisons were performed using Tukey's test where applicable.

^b^ *p* values calculated using the *t*‐test for two‐group comparisons.

The mean values for LAmax, LA:Ao, LVIDd, LVIDs, VMA, GMA, GMV, VMA‐GMA, VLVOT and GLVOT were significantly greater in dogs with mitral regurgitation and cardiomegaly than in those with mitral regurgitation but without cardiomegaly (*p* ≤ 0.05). No statistically significant differences were observed in the variables Ao, IVSd, LVPWd, IVSs, LVPWs, EF, FS, VA and GA among the groups with varying severities of mitral regurgitation and different heart sizes (*p* > 0.05).

Table 3 indicates that there are statistically significant differences in the variables FS, GMV, VLVOT and GLVOT between the clinical and preclinical groups.

**TABLE 3:** Echocardiographic variables in 60 small‐breed dogs with myxomatous mitral valve disease (MMVD), grouped by clinical signs.

Group variable	Abnormal cardiovascular clinical sign (*n* = 60)		
	Preclinical (*N* = 51)	clinical (*N* = 9)	*p* value (*t*‐test)^a^
LAmax (cm)	1.60 (0.78–3.03)	1.95 (1.34–3.08)	0.10
Ao (cm)	1.19 (0.66–1.54)	1.29 1.03–1.48)	0.74
LA: Ao	1.35 (0.84–2.72)	1.50 (0.93–2.18)	0.55
IVSd (cm)	0.38 (0.16–0.55)	0.38 (0.23–0.56)	0.33
LVIDd (cm)	2.63 (1.21–3.87)	3.15 (2.38–4.87)	0.35
LVPWd (cm)	0.38 (0.18–0.59)	0.40 (0.21–0.56)	0.06
IVSs (cm)	0.64 (0.34–0.93)	0.70 (0.41–0.89)	0.72
LVIDs (cm)	1.76 (0.93–2.69)	2.11 (1.53–3.06)	0.61
LVPWs (cm)	0.67 (0.36–2.69)	0.69 (0.47–0.89)	0.72
EF (%)	62.58 (41–80)	62.11 (50–70)	0.12
FS (%)	32.70 (19–47)	32.44 (24–38)	0.05
VMA (cm/s)	65.13 (38.70–111.50)	84.65 (43.10–128.30)	0.13
GMA (mmHg)	2.01 (0.60–11.80)	2.84 (0.50–6.60)	0.35
VMV (cm/s)	58.51 (31.00–121.40)	61.50 (35.40–90.70)	0.13
VMA‐VMV (cm/s)	6.61 (−32.90 to 37.90)	23.15 (−16.60 to 37.60)	0.65
GMV (mmHg)	1.42 (0.40–3.80)	1.71 (0.50–3.30)	0.01
VA (cm/s)	68.19 (41.30–99.60)	64.28 (45.40–104.00)	0.18
GA (mmHg)	2.00 (0.70–4.00)	1.65 (0.80–4.30)	0.40
VLVOT (cm/s)	61.69 (34.30–92.10)	69.72 (44.30–99.30)	0.002
GLVOT (mmHg)	1.56 (0.70–3.20)	2.02 (0.80–3.90)	0.000

Abbreviations: Ao, aorta dimension; EF, ejection fraction; FS, fractional shortening; GA, pressure gradient of aorta; GLVOT, pressure gradient of left ventricle outflow tract; GMA, pressure gradient of mitral flow wave at atrium side; GMV, pressure gradient of mitral flow wave at ventricle side; IVSd, interventricular septum thickness during the diastole; IVSs, interventricular septum thickness during the systole; LAmax, left atrial dimension; LVIDs, left ventricular internal dimension at end‐diastole; LVIDs, left ventricular internal dimension at end‐systole; LVPWd, left ventricular posterior wall thickness at end‐diastole; LVPWs, left ventricular posterior wall thickness at end‐systole; *N*, number of cases; VA, peak velocity of aorta; VLVOT, peak velocity of left ventricle outflow tract; VMA, peak velocity of mitral flow wave at atrium side; VMV, peak velocity of mitral flow wave at ventricle side.

^a^ *p* values calculated using one‐way ANOVA for three‐group comparisons and independent *t*‐test. Post‐hoc pairwise comparisons were performed using Tukey's test where applicable.

^b^ Values are presented as mean (minimum–maximum).

### 3.5 Correlation Analyses

**FIGURE 5:**
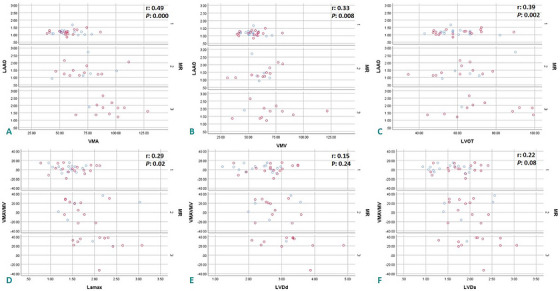
Correlations between echocardiographic measurements in 60 small‐breed dogs with mitral regurgitation (MR) of varying severity: mild (1), moderate (2) and severe (3). All measurements were obtained using brightness (B‐mode), motion (M‐mode) and Doppler echocardiography. Red spots indicate female dogs; blue spots indicate male dogs. (A) LA/Ao ratio versus VMA. (B) LA/Ao ratio versus VMV. (C) LA/Ao ratio versus LVOT. (D) VMA/VMV ratio versus LAmax. (E) VMA/VMV ratio versus LVIDd. (F) VMA/VMV ratio versus LVIDs. Ao, aorta; LA, left atrium; LAmax, maximum left atrium diameter; LVIDd, left ventricular internal diameter in diastole; LVIDs, left ventricular internal diameter in systole; LVOT, left ventricular outflow tract; MR, mitral regurgitation; VMA, mitral annular velocity at atrial side; VMV, mitral annular velocity at ventricular side. *r* = Pearson correlation coefficient. *p* = significance level for correlation (calculated using Pearson correlation test).

As shown in Figure 5, a moderate positive statistical correlation was observed between LA:Ao and three Doppler echocardiographic variables: VMA (*p* = 0.00, *r* = 0.49), VMV (*p* = 0.008, *r* = 0.33) and LVOT (*p* = 0.002, *r* = 0.39). Additionally, a weak but statistically significant correlation was found between VMA‐VMV and LAmax (*p* = 0.02, *r* = 0.29). However, no differences in correlation were observed between these factors and motion‐mode echocardiographic variables.

## 4 Discussion

This study revealed a statistically significant correlation between Doppler hemodynamic variables and MR severity. As MR severity increased, the velocity and gradient variables at the mitral valve and left ventricular outflow tract also increased. One of the earliest studies on echocardiography and MR in dogs was conducted by Braunwald et al. (1957) and their findings align with those of the present study. According to Vezzosi et al. (2021) and Morgan et al. (2020), the mitral valve velocity increases with the severity of MMVD. Similarly, Larouche‐Lebel et al. (2019) demonstrated significant correlations between Doppler echocardiographic variables and different MR severity levels in dogs with MMVD. Hezzell et al. (2012) also reported higher velocities in dogs with severe (fatal) MR than in nonfatal MR cases. Studies by Suzuki et al. (2013) and Thomas et al. (1998) Thomas et al. (1998) support these findings. Additionally, Muzzi et al. (2003) reported a significant correlation between colour flow mapping of the regurgitant jet and Doppler variables in dogs with congestive heart failure.

This study also revealed a significant correlation between LVID in systole and diastole and MR severity. Similarly, Lord et al. (2010), in their study on Cavalier King Charles Spaniels, reported a direct relationship between increased LVIDs, MR severity and the occurrence of congestive heart failure.

The results of this study demonstrated a significant increase in LA:Ao with increasing MR severity. Gouni et al. (2007) conducted a similar study on dogs with varying MR severities and reported significant differences in LA:Ao between groups. Serres et al. (2008) and Larouche‐Lebel et al. (2019) reported similar findings.

According to studies such as Höllmer et al. (2017), left atrial size increases in response to MR. Olsen et al. (2003) reported that in Dachshund dogs, mitral valve prolapse severity, regurgitant jet size and murmur intensity positively correlated with increased LA size over time. These findings align with human studies (Kihara et al. 1988; Yellin et al. 1979). However, some studies, such as Larouche‐Lebel et al. (2019), did not find significant correlations between B‐mode variables and MR severity.

The present study revealed differences in FS, LVOT and GLVOT between clinical and preclinical MMVD patients. Schober et al. (2010) reported that the LA:Ao ratio and velocity flow were significantly greater in clinical MR groups than in preclinical groups. Chetboul and Tissier. (2012) proposed Doppler echocardiographic measurements as valuable indices for evaluating MMVD in preclinical dogs and predicting disease progression. Similarly, studies in humans have shown that velocity variables are significantly greater in clinical MR patients (Al‐Wakeel et al. 2015).

The results of this study indicated that LA size can be used to estimate the severity of the blood jet, but it is not an ideal criterion for distinguishing between clinical and preclinical MMVD patients. Dickson et al. (2017) reported similar findings.

No differences in FS or EF were observed among dogs with varying MMVD severity. However, Gouni et al. (2007) and Suzuki et al. (2013) reported that FS is correlated with disease severity. Vezzosi et al. (2021) measured FS in dogs with late‐stage, severe, moderate and mild MMVD as 51%, 50%, 46% and 39%, respectively, and reported statistically significant differences between each group and the mild/moderate groups. Serres et al. (2008) reported that FS and EF increased with disease severity. Differences in grouping, disease duration and sample size may explain the discrepancies between this study and others. Factors such as preload, afterload and the creation of a pathological low‐resistance pathway by mitral valve lesions can influence these variables, as noted by Boon (2011).

This study revealed that heart size was greater in dogs with severe MR and clinical signs. Similarly, Mihara et al. (2021) reported that the mitral valve annulus was larger and more circular in dogs with MMVD than in control dogs. However, Sargent et al. (2015) argued that MR severity provides prognostic value independent of heart size in MMVD patients.

The findings also revealed that MMVD incidence was greater in intact dogs. No differences were observed in terms of age, weight or sex. Similarly, Suzuki et al. (2013) reported no correlation between age, weight or MMVD incidence. However, Atkins et al. (2009) reported a higher MMVD incidence in male dogs than in female dogs, contrary to the findings of this study. This finding was consistent with the study by Elyasi et al. (2023) in cats. In human studies, postmenopausal estrogen reduction has been linked to adverse cardiovascular effects, including increased blood pressure, sympathetic tone and collagen/elastin deposition in the heart, potentially accelerating mitral degeneration and calcification (Huxley 2007).

## 5 Limitations

In the present study, echocardiographic and clinical evaluations were conducted only at the initial visit. As a result, it was not possible to differentiate cases on the basis of disease duration (i.e., acute vs. chronic). Furthermore, the sample size was limited, highlighting the need for larger population studies in the future.

## 6 Conclusions

The findings of this study indicate that echocardiography, particularly Doppler echocardiography, is a valuable tool for diagnosing and grading MMVD. This study demonstrated that velocity variables in affected patients vary according to the severity of MR, changes in heart size and the presence of clinical signs. Therefore, evaluating these variables allows for more precise treatment strategies and follow‐up care. Additionally, the higher prevalence of this disease in intact dogs highlights the potential impact of neutering on cardiac health and the development of heart disease. Thus, the results of this study can serve as a reference for managing MMVD cases.

## Author Contributions

**Boshra Elyasi**: conceptualization, investigation, writing – original draft, funding acquisition, methodology, validation, visualization, writing – review and editing, software, formal analysis, project administration, data curation, supervision, resources.

## Funding

The author has nothing to report.

## Ethics Statement

This study was conducted on client‐owned animals referred to Dr. Taghipour Veterinary Hospital, Tehran, Iran. All procedures were performed as part of routine clinical practice, and no additional diagnostic or therapeutic interventions were carried out for research purposes. Therefore, formal ethical committee approval was not required. Informed consent was obtained from all pet owners prior to inclusion in the study. Owners were informed that anonymized clinical data would be used for research purposes and may be published as part of a scientific article.

## Conflicts of Interest

The author declares no conflicts of interest.

## Data Availability

The datasets used and/or analysed during the current study are available from the corresponding author and can be provided upon reasonable request.
